# Review of clinical brachytherapy uncertainties: Analysis guidelines of GEC-ESTRO and the AAPM^☆^

**DOI:** 10.1016/j.radonc.2013.11.002

**Published:** 2014-01

**Authors:** Christian Kirisits, Mark J. Rivard, Dimos Baltas, Facundo Ballester, Marisol De Brabandere, Rob van der Laarse, Yury Niatsetski, Panagiotis Papagiannis, Taran Paulsen Hellebust, Jose Perez-Calatayud, Kari Tanderup, Jack L.M. Venselaar, Frank-André Siebert

**Affiliations:** a Department of Radiotherapy, Comprehensive Cancer Center, Christian Doppler Laboratory for Medical Radiation Research for Radiation Oncology, Medical University of Vienna, Austria; b Department of Radiation Oncology, Tufts University School of Medicine, Boston, USA; c Department of Medical Physics & Engineering, Sana Klinikum Offenbach, Germany; d University of Valencia, Spain; e University Hospital Gasthuisberg, Leuven, Belgium; f Quality Radiation Therapy, The Netherlands; g Nucletron an Elekta Company, The Netherlands; h Medical Physics Laboratory, Medical School, University of Athens, Greece; i Department of Medical Physics, Oslo University Hospital, The Radium Hospital, Oslo, Norway; j Department of Physics, University of Oslo, Oslo, Norway; k Hospital Universitario La Fe, Valencia, Spain; l Aarhus University Hospital, Denmark; m Department of Medical Physics and Engineering, Instituut Verbeeten, Tilburg, The Netherlands; n University Hospital S-H Campus Kiel, Germany

**Keywords:** Brachytherapy, Dosimetry, Uncertainties, Treatment planning

## Abstract

**Background and purpose:**

A substantial reduction of uncertainties in clinical brachytherapy should result in improved outcome in terms of increased local control and reduced side effects. Types of uncertainties have to be identified, grouped, and quantified.

**Methods:**

A detailed literature review was performed to identify uncertainty components and their relative importance to the combined overall uncertainty.

**Results:**

Very few components (*e.g.*, source strength and afterloader timer) are independent of clinical disease site and location of administered dose. While the influence of medium on dose calculation can be substantial for low energy sources or non-deeply seated implants, the influence of medium is of minor importance for high-energy sources in the pelvic region. The level of uncertainties due to target, organ, applicator, and/or source movement in relation to the geometry assumed for treatment planning is highly dependent on fractionation and the level of image guided adaptive treatment. Most studies to date report the results in a manner that allows no direct reproduction and further comparison with other studies. Often, no distinction is made between variations, uncertainties, and errors or mistakes. The literature review facilitated the drafting of recommendations for uniform uncertainty reporting in clinical BT, which are also provided. The recommended comprehensive uncertainty investigations are key to obtain a general impression of uncertainties, and may help to identify elements of the brachytherapy treatment process that need improvement in terms of diminishing their dosimetric uncertainties. It is recommended to present data on the analyzed parameters (distance shifts, volume changes, source or applicator position, etc.), and also their influence on absorbed dose for clinically-relevant dose parameters (*e.g.*, target parameters such as *D*_90_ or OAR doses). Publications on brachytherapy should include a statement of total dose uncertainty for the entire treatment course, taking into account the fractionation schedule and level of image guidance for adaptation.

**Conclusions:**

This report on brachytherapy clinical uncertainties represents a working project developed by the Brachytherapy Physics Quality Assurances System (BRAPHYQS) subcommittee to the Physics Committee within GEC-ESTRO. Further, this report has been reviewed and approved by the American Association of Physicists in Medicine.

Brachytherapy (BT) has evolved into a high-technology modality of radiotherapy incorporating 4D imaging and sophisticated planning methods as standards of care. However, clinical BT uncertainties have not been adequately addressed. Often, the technique itself has been described without uncertainties in discussions about dose-delivery accuracy or planning target volume (PTV) and clinical target volume (CTV) margins [1]. It is apparent that if the therapeutic radiation source is brought close to or into the target volume, uncertainties in defining location of the delivery device in relation to the regions of interest are small compared to most external-beam radiotherapy (EBRT) methods. In BT, a source is often placed within the target volume; there are very high doses and dose gradients within this volume. However, the target volume can still be well covered with the prescribed dose.

BT is subject to uncertainties from imaging, treatment planning, dose delivery, and anatomical variations. It is essential to identify these uncertainties, their magnitude, and their impact on the overall uncertainty of dose delivery to the patient. This overall uncertainty can be compared to that available for the most sophisticated EBRT techniques, *i.e*., image-guided radiotherapy (IGRT). Having this knowledge may provide correct dose assessment for BT, dose effect modeling, and subsequently improved clinical outcome when using better planning aims with dose and volume constraints.

While the primary goals of estimating uncertainties in clinical BT are to minimize therapy delivery variations and to improve patient outcomes such as disease-free survival and organs-at-risk (OARs) toxicities, these outcomes cannot be estimated quantitatively in a direct manner for all situations. In most cases, the uncertainties for the delivered physical dose per fraction can be chosen as a surrogate for expected changes in biological response. However, in cases where relationships with biologically weighted total dose values are available, the overall uncertainty can be linked to these total dose values by equi-effective dose with 2 Gy fractionation using the linear quadratic model (EQD2) [2–4]. The purpose of this report is to present a minimum standard for an uncertainty analysis of the clinical practice of photon-emitting BT. The relationship with absorbed dose is sought wherever possible. Identifying uncertainties in the CTV delineation, including inter- and intra-observer variability in target and OAR contouring, is not the goal of this report and these uncertainties are only discussed for comparison.

## Methods and materials

### Uncertainty definitions

The current standardized method for evaluating and expressing measurement uncertainties is based on the Comité International des Poids et Mésures (CIPM) report [5]. This method has been developed further by the International Standards Organization (ISO) in the Guide to the Expression of Uncertainty in Measurement (GUM) as recently updated in 2010 [6] and by the U.S. National Institute of Standards and Technology (NIST) Technical Note 1297 [7]. This method categorizes uncertainties into either Type A (statistical) or Type B (everything else) components. Regarding the uncertainty of an observation, precision is the reproducibility (Type A) of the observation while accuracy is the difference of the observation to the true value.

The magnitude and distribution of an individual uncertainty component should be estimated. The established method for estimating the overall uncertainty is to combine all uncertainty components, expressed as relative standard uncertainties, in quadrature. This may be visualized as Eq. (1) where *s_i_* is the standard deviation of the mean of multiple measurements and is the quadrature sum of all Type A uncertainties, and *u_j_* is the quadrature sum of all Type B uncertainties.

(1) $$V=2\sqrt{\left(s_{i}^{2}+u_{j}^{2}\right)}$$

This approach assumes many degrees of freedom and non-correlated uncertainty components, which is generally the case for clinical BT. In the GUM report, the overall uncertainty is expressed as *U*. However, this nomenclature is not convenient for the field of BT since *U* is the recognized symbol for the units of air-kerma strength. Consequently, the overall uncertainty in this report is expressed as *V* as recommended in the joint AAPM/GEC-ESTRO report on BT dosimetric uncertainties (TG-138) [8]. Further, a 2 is present before the square-root symbol to indicate a coverage factor of two (*k* = 2) as recommended for clinical BT in the TG-138 report. Individual uncertainty components should be estimated and expressed with *k* = 1, with only the overall uncertainty expanded as *k* = 2 toward estimating the uncertainty of an observation for a 95% confidence interval (assuming an approximately normal distribution).

The scope of this report is dosimetric evaluation for clinical purposes, not for regulatory purposes or to identify medical events.

### Literature review

The first step of this project was a joint brainstorming within the GEC-ESTRO BRAPHYQS (BRAchytherapy PHYsics Quality assurances System) experts to identify the different components of uncertainty in BT. Working groups were appointed to collect data for each uncertainty component for gynecological, breast, and prostate BT. Data collection included literature review, internal documents for commercial devices, and published quality assurance (QA) guidelines or recommendations. Examples are provided on the uncertainty type, value, and the reference. Based on this information, a review of known and documented uncertainty components and their magnitude is provided in an effort to define overall uncertainties and, mainly, assess their relative importance. The review facilitated the drafting of recommendations for uniform uncertainty reporting in clinical BT, which are also provided.

## Sources of uncertainty

### General observations

During the literature review, there was a major difficulty obtaining uniform information according to the currently defined uncertainty methodology of Section Uncertainty definitions. In many cases, authors did not demarcate Type A from Type B uncertainties. Uncertainties for technical afterloader parameters, applicators, or organ movements were presented either in the quantities of distances (*e.g.*, mm) or in dose differences due to geometrical shifts. Such dose differences were then reported as absolute values (*i.e*., Gy) or as percentage dose differences (%). Absolute dose values were reported for dose per fraction or the impact of the uncertainties on the total dose. The percentage difference can be related to dose per fraction, total dose from BT, or total dose from EBRT plus BT (such as for combined therapies). Furthermore, all these dose values could be presented as either physical dose or EQD2 dose. Most uncertainties related to dose or volume parameters were based on studies of institutional patient cohorts. This led to differences in uncertainty reporting using, for example, mean or median values and different ways to report variation (standard deviation at *k* = 1, expanded uncertainties at *k* = 2, min/max, etc.). In short, there is no uniform method in practice for reporting the influence of uncertainties on BT dosimetric parameters. Consequently, this report intends to provide a uniform reporting method.

### Source strength

Source strength is a basic parameter prevalent in all BT dose calculation formalisms. It influences all levels of treatment planning, from sophisticated 4D image-based plans to simple plans based on time per source-strength unit taken from tables or dose atlases. Consequently, calculations of BT absorbed dose uncertainties begin with estimations of source-strength calibration uncertainties. Following AAPM Task Group 32 [9], ICRU 38 [10], and IAEA-1274 [11], there is uniform agreement in the BT community to specify source strength for photon-emitting sources in terms of air-kerma strength, either *S*_K_ or RAKR [12,13].

In the jargon of BT, the field may be categorically divided into low-energy (LE) and high-energy (HE) photon emitters, for which 50 keV serves as the boundary for the mean energy of the emitted photon spectrum [14]; and also low-dose rate (LDR) and high-dose rate (HDR) sources, defined as resulting in absorbed dose rates <2 Gy/h or >12 Gy/h, at the dose prescription level, respectively [10]. BT sources may be used in either temporary or permanent implants, an aspect that does not influence source-strength calibration uncertainties.

The most comprehensive report on uncertainties for BT source strength calibration to date is the TG-138 report [8]. This joint AAPM/ESTRO report covers the primary calibration standard, its link to the vendors providing a source-strength certificate, and source calibrations performed at the departmental level. It also examines the relative uncertainties in source-strength calibrations for individual sources and estimates these as 1.3% (*k* = 1) for LE and 1.5% (*k* = 1) for HE (see Tables 1 and 4 in the TG-138 report), except for LE-HDR. While this latter category would include electronic BT sources or HDR ^103^Pd sources where current source calibration methods are under development and uncertainties involved are much larger than the aforementioned values, the literature examined herein did not include these source types.

Individual source strengths and their respective position within an implant are not tracked in LE-LDR BT source implants. LE-LDR sources are typically shipped by the manufacturer with source strengths grouped in batches of ±2% to ±8% bin widths. Assuming a rectangular distribution of source strength within these bins, the calibration uncertainty increases (in quadrature) by $\frac{\sigma }{2\sqrt{3}}$ or to 0.6% and 2.3%, respectively. In combination with the LE-LDR single-source uncertainty of 1.3%, the overall uncertainty with these bin widths increases to 1.4% and 2.6% when accounting for manufacturer source-batching practices as explained in the TG-138 report.

### Treatment planning

The current method of BT dose calculations is based on the AAPM TG-43 dose calculation formalism [15,16]. Reference BT dosimetry parameters are determined in the vicinity of a single source that is centrally positioned in a spherical water phantom of either 15 cm or 40 cm radius, for LE and HE sources, respectively [17]. Treatment planning systems (TPS) incorporate BT dosimetry parameters in a table-based approach where the approximate effect of dose falloff with diminishing solid angle is divided out as the geometry function. While there are uncertainties in the derivation of each of the dosimetry parameters, it is their combined relationship upon TPS calculation of absorbed dose that is relevant to clinical BT. Uncertainty in the calculation of absorbed dose to water in water *D*_w,w_ will vary as a function of source type and location of the point of interest *P*(*r*, *θ*) in polar coordinates, and will include the source-strength calibration uncertainty described above. At *r* = 1 cm along the source transverse-plane *θ* = 90°, the *D*_w,w_ uncertainty is 3.8% and 2.6% for individual LE and HE sources, respectively [8]. These values have been derived by propagation of best practice uncertainties associated with source strength measurements in the clinic (see preceding section), Monte Carlo (MC) dose estimates, and TPS dataset interpolation (see Table 5 in the TG-138 report). These uncertainties generally increase for smaller *r* due to dynamic components and positioning uncertainties within the source capsule and accuracy of the tabulated values at these short distances. Uncertainties will also, more slowly, increase for larger *r* due to the influence of material cross-sections as radiation passes through thicker media with related uncertainties for dose measurement or MC-method dose estimations.

With a conservative LE source-calibration uncertainty (*k* = 1) of 2.7% taken from an 8% wide batch, a LE dose calculation uncertainty (*k* = 1) of 4.9% (differing from the 4.4% listed in Row 5 of Table 5 in the TG-138 report due to batching) is combined with additional uncertainties from subsequent clinically-oriented procedures as described in the following sections. Similarly, for the HE dose calculation uncertainty (*k* = 1) of 3.4% (see Row 5 of Table 5 in the TG-138 report).

Another uncertainties aspect of brachytherapy is fractionated treatments. Non-systematic uncertainties cause different delivered doses per fraction. This results in differing overall uncertainties depending on the number of fractions. Lowering the fraction dose and increasing the number of fractions therefore results in less uncertainty for the overall dose compared to one single observation in one fraction. For random uncertainties the number of fractions *n* results in a factor related to 1/$\sqrt{n}$ for the overall uncertainty. For such calculations, target and organ variations have to be studied in detail for each individual treatment protocol in order to distinguish the different components for the overall uncertainty.

### Heterogeneity effects

Besides other sources of uncertainty associated with input quantities to TG-43-based dose calculation algorithms, Type B uncertainties are also involved due to the principles and assumptions of the TG-43 formalism. These stem mainly from heterogeneities, *i.e.*, disregarding: (a) differences between tissue elemental composition and density relative to water; (b) patient contour and the position of the source(s) relative to the patient/air interface; (c) high-*Z* materials present in the geometry including applicators, shields, or permanent implants, and, to a lesser extent, incomplete radiological physics approximations, *i.e.*, charged particle disequilibrium, dose contribution by beta emissions, etc. A comprehensive body of literature exists on the effect of heterogeneities for specific BT treatment sites/plans, determined using, most commonly, Monte Carlo simulation. This literature has been augmented in view of the advent of clinically feasible dose calculation algorithms capable of taking heterogeneities into account. An extensive literature review is beyond the scope of this work and interested readers are referred to reviews by Thomadsen et al., Rivard et al., and Beaulieu et al. [18–20].

In the context of this work, existing literature serves as acknowledgement that the magnitude of the offset that material heterogeneities introduce relative to TG-43 dose calculations is possibly important. According to the GUM [6], uncertainty components arising from non-statistical effects (Type B) can be included in a combined standard uncertainty evaluation in the form of standard uncertainties from established probability density functions (PDFs) that are based on the current state of knowledge. However, construction of these necessary PDFs is a formidable problem since Type B uncertainty associated with material heterogeneities is strongly dependent on position within the patient. Therefore, heterogeneities effects as reviewed in the following sections are commonly reported as the percentage changes in DVH parameters for the PTV and OARs.

#### Low-energy

In permanent implants of LE sources used mainly for treatment of prostate cancer, the dose delivered from any seed in an implant is affected by the presence of the other seeds. This inter-seed attenuation (ISA) effect depends on radionuclide (*i.e*., photon energy), source encapsulation, seed spacing, or equivalently seed density, which in turn depends on prostate size and seed air-kerma strength. Initial studies using MC simulations of a number of seeds with different spacing [21] or idealized implants [22] reported an effect on conformity and homogeneity indices that becomes negligible for ^125^I seeds spaced >0.5 cm apart. Subsequent studies of the ISA effect were performed using MC simulations in clinical, CT-based, pre- or post-implant geometries with results reported in CTV and OAR DVH parameters employed for treatment plan evaluation [23–26]. Assuming that seeds are coincident with the needle insertion axis and without accounting for other effects such as edema or seed migration, results range between a negligible effect and a CTV *D*_90_ overestimation of 1–5% depending on study design (pre- or post-implant geometry), prostate volume, seed density, and even seed model.

An important effect has also been reported due to the difference of tissue and water in elemental composition and density [23,25,27–29]. It should be noted that tissue composition information is not currently available in routine clinical practice in contrast to tissue density that can be readily obtained from CT imaging data. In a recent comprehensive study, [29] Landry et al. reported, *inter alia*, on the effect of tissue compositional uncertainties and density on four clinical prostate ^125^I seed implants using three prostate compositions taken from the literature, and four clinical breast ^103^Pd seed implants using varying PTV adipose/gland ratio and adipose/gland elemental composition. For prostate, it was concluded that the effect of composition ranges from negligible to an average *D*_90_ increase of 3.2% relative to water, while the use of densities derived from CT data instead of unit density leads to a decrease of *D*_90_ values by 1–2%. For breast, replacing water by a homogeneous tissue of a representative adipose/gland ratio is the primary factor affecting dosimetric accuracy with the *D*_90_ found to increase by 30% on average for a 70/30% adipose/gland ratio due to the lower *Z*_eff_ value resulting in lower attenuation. This is followed by uncertainty in the composition of adipose and mammary gland tissue, leading to a 10% variation in *D*_90_, and the use of breast density from CT data, resulting in a 4% increase in *D*_90_ on average. In view of the above, the authors state that for breast cases tissue composition effects overshadow the ISA effect reported to correspond to a 3% decrease of *D*_90_ [30].

The most comprehensive study for prostate brachytherapy with regard to the aim of the uncertainty project of this work is that of Carrier et al. where MC dosimetry results are reported for a cohort of 28 permanent implant patients [27]. The ISA effect was found to result in a systematic *D*_90_ decrease with an average of 4.0 ± 0.4% (*k* = 1) for a specific ^125^I seed model. The corresponding influence on the OARs (urethra, rectum, bladder) was also a systematic decrease of all relevant DVH indices, subject however to increased variability between patients. Taking tissue composition and density into account led to a systematic CTV *D*_90_ decrease of 2.6 ± 0.2% (*k* = 1) and a non-systematic effect to OARs.

The above results however were calculated using dose scoring to medium as opposed to dose to water in medium used in the study of Landry et al. [29]. It is also important to note that the distribution of the overall effect for the CTV *D*_90_ in the study of Carrier et al. is neither rectangular nor Gaussian [27].

In conclusion, results on the ISA effect and tissue density for LE sources might be considered conclusive while the quantification of tissue composition effects suffers from the lack of a routine clinical method to obtain average data, local tissue heterogeneities (*i.e.*, calcifications) and non systematic effects for OARs.

#### High-energy

Temporary implants are almost exclusively performed using HE HDR/PDR sources with remote afterloading technology so that ISA effects are irrelevant. The increased relative importance of the Compton scattering effect in dose deposition mitigates concern for heterogeneity corrections other than that introduced by tissue density, tissue/air as well as tissue/lung interfaces, and the presence of shields or other high-*Z* materials. More important for HE versus LE sources is the effect of the difference between scatter conditions that ensue from the finite patient dimensions and the spherical geometry used to generate TG-43 BT dosimetry parameters [31].

In gynecological (GYN) and rectal treatments, the effect of neglecting the attenuation and scatter reduction due to the use of high-*Z* materials has been examined in the literature [32–37]. Dose perturbation by partial shielding varies substantially with shield material, shape and size well as location. The most important offset of the dose distribution occurs in the shielded region, but its magnitude is both distance- and position-dependent and is consequently not constant even for a particular applicator. Perhaps most important, diminishment of scattered radiation from the shielded portion of the irradiated volume can noticeably reduce dose in the unshielded target volume. Recent retrospective assessments of HDR ^192^Ir tandem-and-ovoid treatments using a dose calculation algorithm capable of accounting for material heterogeneities have reported a minimal impact on clinical dose parameters [38,39]. Greater differences are only reported in case of radiographic contrast used for packing and mainly for the rectum dose.

In ^192^Ir breast treatments, patient scatter conditions led to an average TPS dose overestimation of 14% at points close to the tissue/air interface using experimental methods [40]. A subsequent study [41] using MC simulation in a mathematical, patient-equivalent geometry for a multi-catheter implant showed that percentage isodose lines greater than 60% of *D*_90_ were not affected by the finite breast dimensions or the presence of the lung. However, TPS based on the TG-43 formalism overestimated lung dose and lower isodose lines at points lying both close to the breast or lung surface and away from the implant. Skin dose overestimation was 5% in the central breast region and within 10% at all other points. This TPS dose overestimation was greater for ^169^Yb than ^192^Ir, reaching 15–30% [42]. A direct comparison of TG-43 based dose calculations to MC simulations in the CT-based geometries of 18 multi-catheter breast patients taking into account four tissue types was included in a study introducing an analytical scatter correction technique [43]. Average results, reported in terms of dose-volume statistics for the PTV, skin, lung, and heart verified the magnitude of the TG-43 dose overestimation (*i.e.*, 2.6 ± 0.6% for PTV *D*_90_, 4.7 ± 1.2%, 5.3 ± 0.9%, 2.1 ± 3.7% for skin, lung, and heart $D_{0.1{\text{cm}}^{3}}$, respectively).

Besides multi-catheter implants for HDR ^192^Ir breast BT, heterogeneities and tissue/material interface effects also affect the dosimetry of treatments using dedicated partial breast irradiation applicators due to the presence of iodine-containing radiographic contrast medium or air, the finite breast dimensions, and the low density of the lung that are not considered by TG-43 based TPS [44–49]. Comparison of TPS and MC calculations in breast/lung phantoms showed that the TPS overestimates prescription dose by 4–10%, depending on the concentration of the contrast medium and source direction in a Mammosite® applicator, owing mainly to scatter conditions rather than attenuation in the contrast medium which contributed 1.0–4.8% for iohexol (Omnipaque™) concentrations 5–15% by volume in a 45 mm diameter balloon. This overestimation can be addressed for ^192^Ir with simple analytical methods, but not for ^169^Yb due to photon spectrum hardening. A dosimetric effect ranging from 3% to 9% has also been reported due to the air in the SAVI® applicator [49].

### Dose delivery

#### Positional accuracy

Dose delivery accuracy depends on the consistency of the patient and delivery device (sources) geometry between treatment planning and treatment delivery. In this context and for the example of temporary implants, geometry includes the position of the source inside the applicator and the applicator position inside the patient in relation to target and OAR structures. The uncertainties depend on the applicator type, applicator design, and the clinical disease site.

Systematic effects of afterloader accuracy can be verified at commissioning and constancy checks. It is possible to calibrate and eliminate systematic shifts resulting only from the afterloader. However, the constancy (precision) of source positioning during treatment remains to be documented. According to manufacturer specifications, a source precision of ±1 mm can be achieved. Source positioning for HDR and PDR ^192^Ir afterloaders has been evaluated based on measurements performed in 33 institutions in the Netherlands and Belgium [50]. The standard deviation for source-positioning offset for 16 HDR ^192^Ir afterloaders and 11 PDR ^192^Ir afterloaders was ±1.0 mm and ±1.1 mm, respectively. Recently, Manikandan et al. reported dwell position uncertainties for a new generation afterloader using a detector array also showing a standard deviation (*k* = 1) of 1.0 mm [51]. All these uncertainties are for straight applicators and transfer tubes. The source path is defined as the set of subsequent source positions that can be reached inside the applicator. Inside a curved applicator, the BT source attached to a drive cable is pushed toward the outer wall of the applicator [52], which leads to a deviation of the source path from the expected path (simulated by the CT, X-ray, or MRI markers) and the source position compared to source movement in a straight applicator. This results in discrepancies between expected and true source positions, which depend on applicator curvature. Discrepancies of up to 2.5–4.5 mm have been measured in the ring applicator [53–55], and up to 5.5 mm in a 33 mm diameter curved plastic applicator [56]. Type B uncertainties can be substantially decreased by appropriate localization and definition of the source path during commissioning and/or treatment planning [57,58]. In most cases, the diameter of the assumed circular source path that best fits to the real positions will be different than the nominal diameters of the source path. The tension effect of the cable can even result in different subsequent source positions than the programed step size or even no change in source position for programed steps of 1–2 mm in some afterloaders [59]. Fortunately, it is possible to minimize many of these uncertainties by digitizing the real source path during applicator commissioning and using this path for treatment planning. This task will be facilitated with reconstruction tools based on recently available applicator libraries [57].

Another source of uncertainties is the source orientation inside the applicator. For straight applicators, orientation is closely aligned to the source path axis. However, the orientation can be different and not tangential to the source path for curved applicators, especially when the source channel is large compared to the source. This effect will influence the dose distribution. A similar effect, but with a smaller impact on clinical dosimetry, has been reported for unknown orientations of loose seeds inside the prostate [24,60]. In general, the dosimetric consequences of source misplacements are similar to applicator misplacements. In both situations, the physical source position is different from that of the treatment plan position. The impact is specific to clinical disease site and is presented in the next sections.

#### Temporal accuracy

Dwell time delivery by the afterloaders has been verified using a specially developed QA tool with a temporal accuracy of 1 ms [61]. Evaluating the accuracy and precision of an HDR ^192^Ir BT afterloader, the authors found that the bias in dwell time can exceed 60 ms and the dwell time associated with the first dwell position unexpectedly differed by 30 ms. Transit dose, tissue irradiation during source movement, is not taken into account in treatment planning. Measurements in a phantom with the source positioned 5.0 cm from the detector and a dwell time range of 0–120 s for Nucletron HDR and PDR ^192^Ir afterloaders [50], showed that the transit dose varied from 0.004 Gy for a 0.04 Gy m^2^/h HDR source to 0.0004 Gy for a 0.004 Gy m^2^/h PDR source. Relative to the planned doses of 0.75 Gy and 0.40 Gy for HDR and PDR afterloaders, respectively, the maximum contribution of the transit dose to the measured dose was 0.5% for HDR and 0.1% for PDR afterloaders. Additional measurements confirmed that the manufacturers of the afterloaders compensate for the effects of interdwell transit dose by reducing the actual dwell time of the source [62]. The dwell time of the source at each dwell position was reduced by the time taken for the source during transit to compensate for the transit dose. For example, for a source step size of 5 mm and a planned source dwell time of 2 s, the measured average dwell time was 1.93 ± 0.01 s with an average transit dose compensation time of 0.07 ± 0.01 s. Uncertainties due to transit dose could also be minimized by taking them into account during treatment planning [63].

Another potential issue is the “rounding error” introduced when exporting the treatment plan to the afterloader control unit or from the control unit to the afterloader itself. The largest rounding effect can occur when the dose distribution is not optimized and all dwell weights are set to unity, depending on the dwell time values. When the dose distribution is optimized, all dwell weights are different and the rounding-off errors more or less average out. In an optimized dose distribution, temporal rounding-off for a prescription dose of 5 Gy dose is usually <1.0%.

### Imaging

Imaging allows registration of the dose distribution to the patient anatomy. This process is mostly used for aligning the applicator and source path or the outer applicator surface and patient anatomy so 3D dose distributions can be matched to points and volumes reconstructed from radiographs (2 orthogonal films, variable angle or other techniques), CT, MRI, and/or ultrasound (US) images. For the case of 3D imaging (CT, MRI, US) volumes are reconstructed most often from 2D contours on sectional slices or volumetric contouring. For DVH calculations the dose distribution has to be calculated within these structures. Computational limitations and assumptions due to the finite slice thickness result in several sources of uncertainties. Depending on the shape and position of structures, standard deviations of 1–5% in phantom configurations have been reported for cumulative DVH parameters related to 2 cm^3^ volumes ($D_{2{\text{cm}}^{3}}$) when using different commercial TPS [64]. However, large deviations for *D*_100_ have been reported for target structures, up to 5–20%, depending on algorithm version and sampling points. *D*_100_ is typically linked to a single voxel within the structure. As proposed for EBRT, *D*_98_ (termed “the near minimum dose”) may be a superior metric [65–67].

For images with tandem/ring and tandem/ovoid applicators in place, Aubry et al. reported mean absolute imaging distortions of 0.4 mm, 0.8 mm, and 0.8 mm or less for CBCT, MR-T1, and MR-T2 images, respectively [68]. For fusion of magnetic resonance spectroscopy images with CT or US studies using a mapping algorithm, Mizowaki and colleagues report 3D-positional errors of 2.2 ± 1.2 mm (*k* = 1) [69]. Kolkman-Deurloo and colleagues presented reconstruction uncertainties using an Integrated-Brachytherapy-Unit (IBU) for head-and-neck cases [70]. For two patients, the reported deviations in total irradiation time were 1.1% and 0.5% without distortion correction of the fluoroscopic images and −0.3% and 0.0% with distortion correction. In particular for head and neck cases, the implanted plastic catheters have loop geometry. This can result in source positioning uncertainty inside the catheter. In a phantom investigation, Kohr et al. showed that positioning uncertainties for 360° loops depend on the loop diameter and might differ among afterloading devices [56]. Offsets of several millimeters were found. Nevertheless, the largest offsets were found for catheter loops having radii less than the system specifications. Moreover, 360° loops are not used in typical patient cases. The impact of reconstruction uncertainties on dose to structures is dependent on clinical disease site. These effects are discussed in the following sections for GYN, breast, and prostate.

## Uncertainties at the patient level

### Generalized uncertainties for gynecological malignancies

With CT-based reconstruction, intracavitary applicator registration is usually excellent (within 1 mm). Dose variations are small (<4%, *k* = 1) between different CT reconstruction methods at clinically-relevant dose points [53]. Even with MRI-based reconstruction, the variations between different reconstruction methods as well as inter-observer variations are limited to 0.5–1.0 mm (*k* = 1) [71]. The consequence of reconstruction offsets depends on the offset direction and the examined organ [72]. Rectum and bladder are the organs most sensitive to these uncertainties with $D_{2{\text{cm}}^{3}}$ changes of 5 ± 1%/mm for applicator reconstruction offsets in the anterio–posterior direction where 90% of the patients have changes <6%/mm. For other directions and for the HR CTV (*D*_90_, *D*_100_) and sigmoid ($D_{2{\text{cm}}^{3}}$), average changes are <4%/mm although individual patients show reconstruction offsets up to 5%/mm. A systematic rotation of the source path by 2.5 mm, *i.e.*, one source dwell step size, may lead to a deviation of the rectum $D_{2{\text{cm}}^{3}}$ of up to 5%, and up to 2% for HR CTV *D*_90_ [58]. Berger et al. [52] as well as Wills et al. [73] found deviations of 5–10% (one standard deviation) for HR CTV *D*_90_ and OAR $D_{2{\text{cm}}^{3}}$ for different reconstruction methods and observers, respectively. In a series with repeated applicator reconstruction, Hellebust and colleagues observed a relative standard deviation of 5.6% for rectum $D_{2{\text{cm}}^{3}}$ [74]. By using the results from Tanderup and colleagues, the deviations found in these studies [52,73,74] could be explained by an applicator shift of approximately 1–2 mm in the longitudinal direction [72]. This is in accordance with the results from Haack et al. [71]. Kubicky et al., who estimated minimum and maximum applicator shifts to be 1.625 mm and 3.25 mm, respectively. They used results from a previous study to translate these shifts into an average dose uncertainty of 1–2% [75], much smaller than the uncertainties found by Tanderup et al. and Hellebust et al. These discrepancies are explained by the fact that the estimation by Kubicky et al. was based on random shifts of 16–18 needles in a prostate implant, not well representing the dose distribution of a GYN intracavitary implant.

#### Applicator stability and organ variations, fractionated treatments

Since the BT dose gradient is steep, small changes in the *relative* position of a structure and applicator could lead to large changes in the DVH parameters. Applicator displacements relative to important anatomical structures (target volumes and OARs) can occur between applicator insertion and treatment delivery or during treatment delivery itself, especially when using LDR/PDR treatments subtending many hours. The type of applicator, fixation method, and vaginal packing are crucial factors determining applicator stability. Stable applicator geometry with movements relative to rectum diodes below 1.2 ± 0.7 mm (*k* = 1) in all directions has been reported during PDR ^192^Ir BT delivery with tandem-ring applicators [76].

De Leeuw et al. analyzed applicator movements relative to bony anatomy during PDR delivery and found geometrical shifts as large as 6 ± 7 mm (*k* = 1) in the posterior direction [4]. Several studies have previously reported such movements related to the bony structures. However, such analyses neglect that the target and organs may also move relative to the pelvic bones. If an intracavitary applicator is fixed to the cervix by packing, applicator movement will follow movement of the cervix and related target volumes. Recent studies are based on 3D imaging in order to assess geometric changes between the applicator and the target volumes or the OARs. Such changes can happen during an implant, in between two implants, or between imaging and dose delivery. The clinical relevance of the geometrical shifts is found by analyzing the change in DVH parameters for the structure of interest.

De Leeuw et al. analyzed the dosimetrical consequences of applicator displacement seen during a PDR ^192^Ir BT fraction and found occasional applicator displacements resulting in considerable target dose changes for individual outliers [4]. Mean changes after 22 h in bladder and rectal doses for 18 applications were 4 ± 12% and 4 ± 23%, respectively [4]. The changes for CTV were on average −1 ± 9%. Dose for the whole treatment, including two PDR ^192^Ir BT fractions, varied by only −0.3 ± 2.8 Gy. While movements relative to the bones showed some influence on the OAR dose variations, no such relation was detected for the CTV.

If one BT plan is applied for several fractions, interfraction variations will increase dose uncertainties. For EBRT, the treatment is usually delivered with more than ten fractions and interfraction variation is often quantified in terms of standard deviations. Ascribing standard deviations to intrafraction motion is not suitable for a BT schedule with ⩽3 treatment fractions. Hellebust and colleagues used this approach for gynecological BT and demonstrated that the average relative standard deviations for 13 series of 3–6 fractions were 15% and 17.5% for $D_{2{\text{cm}}^{3}}$ for the rectum and bladder, respectively [74]. They did not examine the total treatment course at the individual patient level. However, such analysis was performed by Kirisits et al. and Beriwal et al. [3,77]. Kirisits et al. compared individual MRI-based 3D treatment planning for each of four fractions with the use of only one MRI treatment plan for 14 patients. They found significant mean differences relative to the BT dose (not including EBRT) of 14%, 9%, 22%, and 28% for HR-CTV *D*_90_, bladder $D_{2{\text{cm}}^{3}}$, rectum $D_{2{\text{cm}}^{3}}$, and sigmoid $D_{2{\text{cm}}^{3}}$, respectively. On an individual patient level, much larger differences were seen. For example, the single-plan method would have resulted in an increased sigmoid dose of more than 24% for seven patients. However, this analysis was based on patients with BT applications throughout the EBRT course, resulting in substantial target volume shrinkage across the BT applications. Mohamed et al. performed the same kind of analysis in a treatment schedule with intracavitary PDR ^192^Ir BT starting in the last week of EBRT or following EBRT after most tumor shrinkage had occurred [78]. They found no significant differences between the single-plan method and repeated planning for the majority of parameters. However, there were considerable individual variations of 1 Gy to 3 Gy for total HR CTV *D*_90_ and OAR $D_{2{\text{cm}}^{3}}$ (EQD2) that indicates that reporting of mean values is not sufficient in plan comparison. Beriwal et al. performed a similar analysis [77], but compared treatment planning based on only MR acquisition to treatment planning based on MR for the first fraction and then CT for the consecutive fractions. Analyzing the average DVH parameters, they did not find significant differences between these two methods. The range of differences was not presented and it is therefore difficult to comment on individual patient variations.

Lang et al. investigated the consequences of using one MR acquisition per insertion (delivering two fractions) instead of performing MR imaging prior to each fraction [79]. They found that systematic differences in the mean total EQD2 dose including EBRT were small (usually below 1%) for target and OAR dose parameters. However, the statistical uncertainties (*k* = 1) reached 3% for HR CTV *D*_90_, and up to 6% for OAR $D_{2{\text{cm}}^{3}}$. In total dose, this corresponds to $D_{2{\text{cm}}^{3}}$ dose reporting uncertainties of 4.7 Gy. The uncertainty magnitude varies with small, medium, and large variations for the target, rectum, and both the bladder and sigmoid, respectively. However, care is required to draw general conclusions from these data. This study used a specific treatment schedule, did not have a fixed protocol for bladder filling, and employed a specific method for applicator placement and fixation by packing. Furthermore, while the uncertainties for a single fraction applying a treatment plan based on a single MRI for the first fraction are 12% for *D*_90_ and 23% for $D_{2{\text{cm}}^{3}}$ for the highly-mobile sigmoid, this is compensated by summation of several BT fractions and addition of the EBRT dose.

In a study comparing 10 CT- and MRI-contoured cervical patients Viswanathan et al. [80] found that CT-based delineation of the OAR is sufficient. On the other hand they reported CT-based tumor contours are overestimating the tumor width. In particular a statistically significant difference for the MRI-based *D_90_* with 5.6 Gy. vs. 4.6 Gy for the CT-based *D_90_* of the HR CTV was found.

Nesvacil et al. [81] compared interfraction variations for 123 patients from different centers, who observed effects of OAR motions occurring during BT on the $D_{2{\text{cm}}^{3}}$ (physical dose) using fixed BT plans and two image sets taken at different timepoints (intervals ranging from 5 h to 22 days). No systematic correlation between the relative change of $D_{2{\text{cm}}^{3}}$ and the time interval between observations was found. For OAR, standard deviations (*k* = 1) for differences between two single situations (either applications or fractions) was above 20% and around 13% for HR CTV *D*_90_. However, the overall uncertainty is reduced similar to the single center experience described above for HDR fractionated treatment and multiple PDR pulses.

Mikami et al. investigated applicator displacements during interstitial GYN BT using cylinders and/or perineal templates. Due to the long distance of the needles in tissue, there is a displacement of the needle tip, mainly to the caudal direction. [82]. While the mean displacement is only 1 mm within 21 h, outliers of up to 12 mm were observed.

Some of the studies mentioned above found large interfraction variations while others did not. The analyses performed and the data presentation deviate considerably among studies; this makes comparisons difficult. However, one common finding is seen – some large deviations are observed for individual patients, even if the mean values do not exhibit large variations. Another important observation is that all the analyses are based on new contouring and applicator reconstruction in a second image set. In particular, contouring uncertainty can affect the DVH parameters and consequently the study results and conclusions.

Calculation of cumulative OAR doses from succeeding BT fractions relies currently on addition of $D_{2{\text{cm}}^{3}}$ values for subsequent BT fractions and not on a summation of dose values in individual voxels. This approximation can lead to OAR dose overestimation when different organ parts are exposed to a high dose in different fractions. For the bladder, the effect on $D_{2{\text{cm}}^{3}}$ has been shown to be minor with a median overestimation of 1.5%, whereas the $D_{0.1{\text{cm}}^{3}}$may be overestimated by 11% (EQD2) [83].The same conclusion regarding a “worst case assumption” is expected to apply for the rectum due to limited organ mobility, whereas dose overestimation to the sigmoid might be clinically relevant, although this has not been rigorously assessed.

Finally, the total dose for most gynecological treatments results from a combination of BT and EBRT. The current standard is to take the EBRT dose that is representative of the homogenous dose plateau, and add it to the BT fractions. This dose is usually the prescribed dose to the ICRU 50 point or *D*_50_ for IMRT. Van de Kamer et al. investigated the uncertainties that are introduced by such a direct adding of the parameter values [84]. Without any additional EBRT boost, additively accurate results can be expected. However, any form of boost that is not taken into account by proper voxel-wise dose summation or sufficient surrogate methods can underestimate HR-CTV *D*_90_ by up to 10 ± 6.2%, especially lymph node boosts, and even more pronounced for the parametrium. For parametrial boosts, there are significant uncertainties related to evaluation of total dose to the target and OARs. Fenkell et al. [85] critically evaluated the assumption that the midline block fully protects OARs (bladder, rectum, and sigmoid) while delivering full parametrial boost dose to target regions not covered by intracavitary BT dose. However, they concluded that this assumption was not valid. The parametrial boost contributed 30–40% of the prescribed parametrial boost dose to $D_{2{\text{cm}}^{3}}$ in OARs while only improving the *D*_90_ of HR CTV and IR CTV by 50–70% of the prescribed parametrial boost dose.

### Generalized uncertainties for breast malignancies

Modern multi-catheter breast techniques are normally accomplished using an HDR or PDR ^192^Ir afterloading unit. After general or local anesthesia, stainless steel needles are placed free-hand or template-based into the breast tissue with inter-catheter spacing of about 1.0–2.0 cm in two to three rows. These needles can be replaced by flexible plastic tubes and secured with special buttons at the patient’s skin surface. Catheter reconstruction can be performed either by orthogonal films or in a modern approach using CT as the basis for the 3D treatment planning process. In many cases, the target region (*i.e.*, the lumpectomy cavity) is marked by surgical clips to identify the target volume.

In a combined phantom/patient study, Hensley et al. [86] reported a CT-based catheter reconstruction uncertainty of 1.1 mm to 2.8 mm. Further they found that larger uncertainties can occur due to patient movements while the CT scanning is in process. These findings are related to a relatively slow CT scanner, so that breathing artifacts and partial volume effects are included in the catheter reconstruction. Nevertheless, Aristei et al. [87], Cuttino et al. [88], and Das et al. [89] showed the benefits of 3D-CT based catheter reconstruction over 2D-techniques. A cohort of 50 consecutive patients underwent CT-based treatment planning and dosimetric results were compared against conventional orthogonal film dosimetry using the Manchester system. CT-based treatment planning showed excellent visualization of the lumpectomy cavity, normal structures and better target volume delineation and coverage were achieved. Dwell times of the CT-based technique were in ±7% agreement to those of conventional plans. Cuttino et al. observed PTV *D*_90_ increased from 89% to 95% (*p* = 0.007) when changing from a 2D technique to a CT-guided technique. Moreover the dose homogeneity index showed an increase from 0.77 to 0.82 after changing to the 3D technique (*p* < 0.005). On the other hand, Kolkmann-Deurloo et al. presented accurate results in a study performing reconstructions by using an IBU [70], with average reconstruction offsets of 0.6 mm without distortions correction and 0.2 mm with correction for 25 cm images from a radiograph-based device.

In the book by Wazer and colleagues [90], Das and Thomadsen mentioned that catheters can shift in the breast, and there is no easy way to adjust the relative source positions to the target. Margins in the direction of the catheters must include this uncertainty. A 2 cm CTV-to-PTV expansion is suggested for the overall uncertainty.

Contouring uncertainties for breast BT were studied in the work of Landis et al. [91]. The lumpectomy cavities of 33 patients were evaluated. The cavity, including a 1.5 cm margin, was defined as target volume in a CT dataset by four experienced physicians. The mean PTV was 215 cm^3^, with volumetric variations ranging from 7% to 42% per patient depending on the visibility of the lumpectomy cavity. In a similar study, Petersen et al. presented the conformity index (CI) for a patient group of 30 and three observers [92]. The CI is the ratio of overlapping volume and the encompassing delineated volume – in this case for the target volume. A mean CI value of 0.61 (0.27–0.84 range) was observed.

Interfraction variations were investigated by Kim et al. using the MammoSite balloon applicator [93]. In a study of 19 patients with 10 fractions and CT imaging obtained before each fraction, they found that interfraction variations were patient specific and not clinically relevant for targets and OARs, but maximum variations could be clinically meaningful. The average trapped air gap volume was reduced from 3.7 cm^3^ following implant to 0.8 cm^3^ at time of first treatment fraction. MC calculations and phantom investigations in MammoSite balloons were performed by Kirk et al. to evaluate dose perturbations by contrast media [45]. They found heterogeneity correction factors of 0.99 and 0.98 for 2 cm and 3 cm balloon diameters, respectively, using 6% by volume of contrast solution.

Plans of 15 MammoSite patients were analyzed by Todor et al. [94]. They reported that respiratory motion, tip location mismatch, and daily source position variation are the main factors for dose delivery uncertainty. Due to respiratory motion, balloon volume can be underestimated by 8% and 16% for CT and CBCT, respectively. The *D*_95_ coverage decreased when the applicator tip was not delineated properly. When using small balloons (about 35 cm^3^) a 3 mm uncertainty resulted in a *D*_95_ coverage decrease from 96.3% to 92.6%. For a large balloon (about 99 cm^3^) *D*_95_ coverage decreased from 98.7% to 79.3%. However, it is not obvious why the variations with the large balloon are larger than with a small one. These are the only published data on this type of uncertainty. With a daily source position variation of 1 mm, *D*_95_ coverage decreased from 98.7% to 88.2% over the course of 10 fractions for the 99 cm^3^ balloon.

### Generalized uncertainties for prostate malignancies

Besides contouring uncertainties, source positioning relative to the target and OARs is the main source of uncertainty for prostate dosimetry. For permanent seed implants, the target volume changes substantially during the time of relevant dose delivery. Prostate swelling compared to day-0 volume varies between 30% and 90%, and then edema resolves exponentially with a mean half-life of 9.3 days [95,96]. Post-implant edema is usually not considered in the pre-procedural plan dose calculations. However, edema may have a considerable impact on the delivered dose. The impact of edema on prostate dosimetry was modeled by Yue et al. [97]. They found that for a set of assumed edema parameters (50% volume increase and 10 day volumetric half-life), static ^125^I or ^103^Pd pre-procedural plans overestimate total dose by about 5% and 12%, respectively.

The variation of permanent prostate BT dosimetry as a function of seed localization uncertainty was investigated for ^125^I implants by applying Gaussian noise with standard deviations ranging from 0.5 mm to 10 mm to the seed coordinates of post-procedural plan datasets [98]. The results demonstrated that <5% deviation of prostate *D*_90_ can be expected when seed localization uncertainty is 2 mm, whereas a seed localization uncertainty of 10 mm yielded an average *D*_90_ decrease of 33 Gy. Because of its lower energy and higher dose gradient, the magnitude of seed localization uncertainty is higher for ^103^Pd implants, and the opposite is true for ^131^Cs implants due to their higher energy and lower dose gradient.

In a multi-institutional study by the GEC-ESTRO BRAPHYQS group, Siebert et al. analyzed the impact of CT parameters on seed localization variations [99]. Seed reconstruction accuracy was not dependent on scanned field-of-view, tube current, tube peak voltage, and scan type (axial or spiral). They found CT seed reconstruction accuracy decreases in the longitudinal direction when slice thickness or table index (pertinent pitch for helical scans) >4 mm. For example, using a 2 mm axial slice thickness and table index had a 0.2 mm uncertainty whereas a 5 mm slice thickness and table index had a 0.7 mm uncertainty. Moreover, the quality of seed reconstruction can be dependent on seed model when using seed-detection software. In a phantom test pattern consisting of 9 dummy sources, 0–3 mismatches occurred [99]. A similar phantom study was published by DeBrabandere et al.*,* but included MRI imaging [100]. They inserted 60 dummy seeds into a newly designed gel-based phantom and performed CT and MRI scans. Seed reconstruction uncertainties for 3 mm, 4 mm, and 5 mm thick slices were smaller for CT than MRI (1.5 T) datasets, being 0.9 ± 0.6 mm, 0.9 ± 0.6 mm, and 2.1 ± 0.8 mm for CT and 2.1 ± 1.4 mm, 1.6 ± 1.2 mm, and 1.9 ± 0.9 mm for MRI, respectively.

A further study by the GEC-ESTRO BRAPHYQS group compared uncertainties introduced by contouring, seed reconstruction and image fusion for three patients [101]. Interobserver variation for these three aspects was investigated for post-procedural planning dosimetry using CT alone, T1 and T2 weighted (fused), and CT and T2 weighted (fused) MRI datasets. The largest standard deviations were found for contouring and fusion. The impact on *D*_90_ was highly technique dependent: for contouring, the standard deviations (*k* = 1) were 23%, 18%, and 17% for CT, T1 + T2, and CT + T2, respectively. The *D*_90_ uncertainties for seed localization were smaller, 2%, 7%, and 2% for CT, T1 + T2, and CT + T2, respectively. Fusion uncertainties in *D*_90_ were 6% and 16% for T1 + T2 and CT + T2, respectively.

In an extensive study, Lindsay et al. demonstrated that contouring uncertainties and seed localization uncertainties have a large impact on the predicted radiobiological outcome for LDR ^125^I prostate seed patients [102]. They observed that the largest impact on dosimetry was found to be seed localization uncertainties (6 mm) resulting in changes of the *D*_90_ of the prostate of more than 10%. The mentioned maximum 6 mm uncertainty of seed localization is, compared to other studies, very conservative. Nevertheless it must be pointed out that this was a simulation study, *i.e*. the uncertainties are of a theoretical nature and do not account for uncertainties in radiobiological parameters.

For HDR ^192^Ir BT, substantial changes of the implanted needles relative to the target geometry have been reported between individual fractions when a single implant and CT-based planning is used. A classic report on this topic demonstrated the shift of the interstitial needle tips in the direction of the prostate apex [103]. Without any corrective measures, the mean prostate *D*_90_ dropped from 96% to 64%. This effect was reported by several other groups, also with substantial OAR dose uncertainties [104–107]. Either a cranial PTV margin is needed and/or imaging should be performed for each fraction followed by correction of the dwell time distribution or the needle positions. Another solution is needle design improvement for better needle fixation to the target. Instead of affixing needles to a template outside the body, needle tip fixation inside the target volume is a promising alternative. The mean absolute displacement for such self-anchoring needles during a 3-day PDR ^192^Ir, prostate BT treatment was 1.2 mm [108]. However, mean absolute differences are not directly comparable to the systematic mean difference and the distributed standard deviation.

Different from the experience with CT-based dosimetry, a 3D US-based treatment protocol was tested by performing imaging for the treatment plan plus two 3D US scans close in time to dose delivery [109]. Although a systematic change was observed, the average needle displacement was 1 mm. These geometric changes result in only 1.6% *D*_90_ reductions. However, some parameters were influenced by up to 5% in individual patients. A possible conclusion is to limit applicator displacement and other geometric variations to ⩽1 mm. Not only is displacement of the implanted applicator of interest, but also is the uncertainty in needle locations (particularly the needle tips). In phantom investigations, Siebert et al. showed that US-based needle tip localization in the sagittal view was accurate within 1 mm, yet subject to inter- and intraobserver variations in manual needle tip detection [110]. Further, lower US system gain settings (−15 dB compared to 15 dB) reduced intraobserver variations. Peikari et al. found needle localization variations of 0.5–4 mm, depending on needle position with respect to the US fields and settings of gain and power [111]. The influence of geometric uncertainties on dosimetry was evaluated in a model-based simulation [112]. Source positioning along the catheter and catheter reconstruction uncertainties of 1.5 mm and 1.0 mm, respectively, led to <2% dose uncertainty for dose volume parameters related to lower doses than 200% of the prescribed dose and reaching up to 5% dose uncertainty at locations within a couple millimeters of the catheters. These uncertainties influenced PTV dose and urethra DVH results by <1%. Catheter-reconstruction uncertainties of 2 mm caused dose uncertainties of 2–9% for locations inside the 150% isodose contour.

### Example uncertainty estimates

The three disease sites discussed above are examined in the following examples of dosimetric uncertainty estimation. Uncertainty components are ordered in a quasi-chronological fashion. These dosimetric uncertainties correspond to *k* = 1 typical values that can be achieved with current technology. If not otherwise stated AAPM TG-43 formalism was used for dose calculation. Researchers should report their own findings and identify means of minimizing the largest prevalent dosimetric uncertainties. The inter- and intrafraction changes between imaging and dose delivery need special attention to quantify resultant dosimetric uncertainties – this is a ripe area for research. In common to these uncertainties is the bias with inter- and intra-observer variations in contouring. Contouring uncertainties influence both inter- and intra-observer variations. Any study delineating an organ or a CTV on two image sets includes these variations in the overall results. Interobserver variations have been reported to influence *D*_90_ or $D_{2{\text{cm}}^{3}}$ values by several percent [113] as is the case with intraobserver variations on target volume dose parameters [114]. Assuming normal distributions for the interfraction and intraobserver (i.e., contouring) uncertainties, with no interrelationship, the square of the total uncertainty will correspond to the sum of the squares of the interfraction and contouring uncertainties. As one cannot directly assess the interfraction uncertainty, it may be derived from the square root of the difference between the squares of the total and contouring uncertainties. Analysis of this bias in any clinical study (including contouring) has not been reported so far for BT. Therefore, the given values for interfraction effects could slightly overestimate the real uncertainties given their inclusion of the intraobserver component [115]. The total dosimetric uncertainty is also given with *k* = 1. The major contribution follows from contouring and intra/interfraction variations. For these kinds of uncertainties, the normal distribution is not true for large values. Anatomy limits the possible situations, *i.e*., during fractionated treatment an organ usually cannot get closer to the implant that is in the direct vicinity of the target volume.

#### HDR ^192^Ir GYN vaginal brachytherapy

In this example, Table 1, the relative dosimetric uncertainty is assessed at a point on the transverse plane on the surface (or at 5 mm depth) of a 30 mm diameter vaginal cylinder BT applicator (*e.g*., the prescription point). Dwell times over the 50 mm active length are optimized to cover the lateral cylinder-surface uniformly along the irradiated length with no consideration for dose uniformity on the applicator dome.

#### HDR ^192^Ir GYN cervical intracavitary brachytherapy

In this example, Table 2, the HR CTV *D*_90_ uncertainty for cervix cancer treated by intracavitary HDR ^192^Ir BT is analyzed. The uncertainty for a single fraction is different to the overall *D*_90_, which is expressed in EQD2 for the entire treatment, consisting of EBRT plus several fractions of BT. Delivering the dose in several fractions usually results in smaller variations due to compensating effects in case of random uncertainties.

Assuming 4 fractions with non-systematic organ changes in-between, the uncertainties for dose delivery and interfraction variations are only half (1/√4) of the values stated in Example 2, while the others stay constant as source strength calibration and dose planning is not repeated for subsequent fractions. This would reduce the overall uncertainty to <7%. If we further assume that in a schedule with HDR fractions, with additional 3D imaging prior to each fraction in order to detect anatomic variations, a 5% overall HR CTV uncertainty is obtained.

#### HDR ^192^Ir breast balloon brachytherapy

In this example, Table 3, the relative dosimetric uncertainty is assessed at a point on the transverse plane 10 mm from the surface of a 50 mm diameter multi-lumen breast BT applicator (*e.g*., the prescription point). The dwell position/timing optimization is complex, but largely restricted to the balloon center.

#### LDR ^125^I prostate permanent brachytherapy

In this example, Table 4, the prostate *D*_90_ uncertainty is assessed for a 50 cm^3^ prostate volume (post-implant CT-based on day 0) with intraoperative treatment planning using 104 seeds spaced with a TRUS template.

#### HDR ^192^Ir prostate US-based brachytherapy

In this example, Table 5, the relative dosimetric uncertainties are considered with respect to the *D*_90_ and *V*_150_ dose-volume parameters for the prostate gland (*i.e.*, PTV).

US imaging without silicone-based stand-offs should be performed for better sound coupling to rectal tissue or else there may be *D*_90_ or *V*_150_ errors of 4% and 24%, respectively [116]. Water or US-gel filled balloons should be used for achieving overall geometry reliability of up to 1.0 mm (0.3–0.7 mm range).

A reconstruction accuracy of 0.7 mm is possible for catheters tips when the catheter free-length measurement method (available in both Oncentra Prostate by Nucletron/Elekta and Vitesse by Varian Medical Systems) is used [117]. Since catheter tip accuracy defines the accuracy of source dwell positions generated by the TPS, this is considered as the determinative uncertainty for catheter reconstruction for US based planning. Assuming further a source positioning uncertainty of 1.0 mm from the afterloader for straight catheters [50,51], the total dose calculation uncertainty considering catheter-anatomy reconstruction can be <2% when prostate *D*_90_, *D*_100_, *V*_100_, and *V*_150_ are considered [112]. In general, the published data used for the treatment delivery uncertainty cover the use of metallic catheters and representative prostate volumes in the range 17–60 cm^3^ [109].

For each implant, it is assumed that either a single treatment plan is generated and a single fraction is delivered, or (for the case of delivering more fractions with the same implant) a new 3D US image acquisition is made and a new treatment plan is calculated based on this newly acquired image set. The procedure also requires that patient remains in the same lithotomic position on the operation table until BT delivery is finished. Additionally, the US-probe location with the transversal detector at the base plane remains unchanged until irradiation is complete. Any US-probe manipulation causes further uncertainties, which are unpredictable and not considered in the current analysis [118]. For target contouring uncertainty the use of US imaging is comparable to MRI and therefore not resulting in higher uncertainties compared to CT based delineation [119].

## Summary

The term ‘uncertainty’ in clinical BT is related to prescribing and reporting treatment parameters. The main parameters are dose at certain points or dose to certain volumes. While a prescription or a treatment plan report states these dose values, the dose actually delivered is different for a variety of reasons. Therefore, the interesting result of any study on uncertainties is its impact on the estimation of the delivered dose. However, only a few types of uncertainties (source strength and afterloader timer) are independent of clinical disease site and location of administered dose. Uncertainties, even for a purely water-equivalent geometry, are related to the geometry of the BT implant, in particular the source distance to the regions of interest. These dosimetric uncertainties are more pronounced if heterogeneous tissue composition and/or applicator materials are considered, especially for LE sources. For HE-sources, scatter conditions, such as for the case of skin for breast BT, are of particular relevance. The total dose uncertainty is thus dependent on the clinical disease site and implant geometry. Therefore, it is not possible to perform generalized quantitative ranking of these uncertainties – only broad statements can be made or general examples based on disease site.

One should strive to identify and minimize systematic (Type B) uncertainties. These include the following items:

(1) Imaging uncertainties due to potential magnification or reconstruction uncertainties. This includes global shifts and systematic differences between the source path used for planning and the often improperly identified source path and source orientation during dose delivery due to inaccurate 1st dwell positions or ring applicator internal cavity.
(2) Treatment planning uncertainties related to contouring the target and OAR.
(3) Treatment planning uncertainties such as deviations of accepted TG-43 parameters from consensus data or due to interpolation or model simplifications. Neglect of systematic shielding effects due to applicator materials is an error, not an uncertainty.
(4) Dose delivery uncertainties due to temporal rounding off or systematic offset in source positioning and dependence on catheter curvature.

A priority ranking of uncertainties other than the above-mentioned systematic ones is as follows. From the findings within this report, the largest uncertainties are at the patient level and related to anatomical differences between the real patient situation at the time of dose delivery and the planned patient geometry and organ definitions. Such uncertainties can become larger with increasing time between the treatment planning imaging and dose delivery, which is the case in fractionated treatments. However, there is no evidence that this trend always holds true. Sometimes uncertainties become smaller over time, as for example with edema resolution following permanent prostate BT. Additionally, there is no evidence for GYN BT that there are larger uncertainties with PDR than with HDR treatments.

However, here uncertainties and variations have to be distinguished. For specific treatment approaches, there are sometimes systematic geometry variations, *e.g*., by applicator displacement or by the target volume changes. Systematic variations due to edema or tumor shrinkage could be taken into account in the overall dose assessment if they can be quantified accurately. In most cases, such variations can also be visualized and tracked by imaging devices or a suitable *in vivo* dosimetry tool and corrected. Here, we have a strong link to resource management. How often, how accurate, and with which kinds of imaging devices do we have to check the BT treatment geometry? There is no evidence with EBRT, including advanced procedures such as SBRT (stereotactic body radiotherapy), that image-guidance for each individual fraction is necessary for all disease sites. Similarly in BT, while imaging and adaptive treatment planning seem beneficial for each fraction and for certain approaches, the consequences of dosimetric uncertainties due to patient variations are not clear. If certain threshold levels for tumor control or limitation of side effects are known, it is possible that dose deviations of several percent will not influence the clinical outcome (*e.g.*, the very high doses applied within *V*_100_ for gynecological and prostate treatments). If doses are small compared to those associated with side effects, uncertainties beyond several percentages may be ignored without major consequences. Consider the example of an organ far from relevant isodose lines compared to an organ in direct vicinity to the implant and high doses. In addition, the spatial dose distribution could remain as a key element for outcome. If a certain part of a target volume is already just covered with the appropriate dose level, and uncertainties lead to dose reduction in this specific region, the impact on local control could be much higher compared to some variation in the overall *D*_90_.

### Recommendations for uncertainty reporting

Most reports of BT uncertainties are not presented in a systematic manner. Clear guidelines are needed for authors presenting their studies in the literature, and should include uncertainty estimates. Every study on uncertainties in clinical BT should report its results to allow reproduction and further comparison with other studies. Therefore the following basic requirements are needed:

(a) Clearly distinguish between uncertainties, variations (which can be quantified), and errors and mistakes.
(b) Quantify Type A and Type B uncertainties as outlined in the TG-138 report, and describe the approach taken to quantitatively ascribe values.
(c) Use a *k* = 1 coverage factor for individual uncertainty components when reporting the standard deviation in addition to any mean value.

The study results should contain data on the analyzed parameter (distance shifts, volume changes, source or applicator position, etc.), and also be expressed with their impact on absorbed dose for clinically-relevant dose parameters (*e.g.*, target parameters such as *D*_90_ or OAR dose). Additionally, this reporting should include total dose uncertainties for the entire treatment course (including several fractions and EBRT dose if applicable) and in terms of equi-effective or isoeffective dose as EQD2 [66,67].

The use of EQD2 dose reporting allows no direct application of the uncertainties related to the absorbed dose reporting. The EQD2 calculation strongly depends on the dose level due to the non-linear calculation. High dose levels with large uncertainties cause pronounced uncertainties for EQD2 dose, with non-symmetric deviations around the reported values. For boost treatments, the total dose uncertainty becomes smaller compared to the uncertainty of only the BT dose because it only partially contributes to the total dose.

### Conclusion

Understanding the contributing factors to the overall uncertainty permits uncertainty reduction and improvements in treatment delivery. The field of BT dosimetric uncertainty is an important topic in need of additional research, combining clinical results with a methodological assessment of how the treatment occurred. With the established method on how to report BT dosimetric uncertainties, attribution of resources to minimize these uncertainties may result in improved treatment planning, patient imaging, and treatment delivery techniques toward better inter-institutional consistency and overall clinical outcomes.

## Potential conflicts of interest stated by the authors

Department of Radiotherapy at Medical University of Vienna receives financial and/or equipment support for research and educational purposes from Nucletron, an Elekta company and Varian Medical Systems, Inc. Christian Kirisits is a consultant to Nucletron, an Elekta company.

Dimos Baltas is a member of Advisory Board and Consultant for Eckert & Ziegler BEBIG GmbH.

Facundo Ballester and Jose Perez-Calatayud received research funding from Nucletron, an Elekta company.

Yury Niatsetski is an employee of Nucletron, an Elekta Company.

Jack L.M. Venselaar is a consultant to Nucletron, an Elekta company.

## Figures and Tables

**Table 1:** Example 1 – HDR ^192^Ir BT source for vaginal cylinder applicator.

Category	Typical level (%)	Assumptions
Source strength	2	PSDL traceable calibrations
Treatment planning	3	Reference data with the appropriate bin width
Medium dosimetric corrections	1	Valid for applicator without shielding and if CTV located inside pelvis; an advanced dose calculation formalism should be used if this assumption false
Dose delivery including registration of applicator geometry to anatomy	5	Accurate QA concept for commissioning and constancy checks, especially for source positioning and applicator/source path geometry, appropriate imaging techniques (either small slice thickness, 3D sequences or combination of different slice orientations), applicator libraries (either by using software solutions or manual)
Interfraction/Intrafraction changes between imaging and dose delivery	5⁎	For one treatment plan per applicator insertion and measures to detect major variations for subsequent fractions
Total dosimetric uncertainty (*k* = 1)	**8**	For treatment delivered with the same BT source

⁎ Estimated value based on expert discussion.

**Table 2:** Example 2 – HDR ^192^Ir source for intracavitary, image-guided cervical cancer BT.

Category	Typical level (%)	Assumptions
Source strength	2	PSDL traceable calibrations
Treatment planning	3	Reference data with the appropriate bin width
Medium dosimetric corrections	1	Applicator without shielding and CTV inside pelvis (concerning for scatter); an advanced dose calculation formalism should be used if this assumption false
Dose delivery including registration of applicator geometry to anatomy	4	Accurate QA concept for commissioning and constancy checks, especially for source positioning and applicator/source path geometry, appropriate imaging techniques (either small slice thickness, 3D sequences or combination of different slice orientations), applicator libraries (either by using software solutions or manual)
Interfraction/Intrafraction changes *(including contouring uncertainties)*	11	For one treatment plan per applicator insertion but several subsequent fractions – if only one fraction is applied the remaining uncertainty between imaging and dose delivery should be at least smaller than this interfraction variation
Total dosimetric uncertainty *(including contouring uncertainties)* (*k* = 1)	**12**	For treatment delivered with the same BT source – note that in cervix cancer BT, both HDR and PDR schedules consist of several fractions, reducing the random uncertainties (see text)

**Table 3:** Example 3 – HDR ^192^Ir BT source for breast balloon applicator.

Category	Typical level (%)	Assumptions
Source strength	2	PSDL traceable calibrations
Treatment planning	3	Reference data with the appropriate bin width
Medium dosimetric corrections	3	Balloon filled with standard level of contrast agent, no consideration or composition of chestwall, lung, or breast
Scatter dosimetric corrections	7	A non-scalar correction for skin dose (and at points in proximity to the surface near the balloon) is needed, and will require an advanced dose calculation formalism to properly account for radiation scatter conditions in the patient. Use of a single prescription point might be not sufficient
Dose delivery including registration of applicator geometry to anatomy	7	Accurate QA concept for commissioning and constancy checks, especially for source positioning and applicator/ source path geometry, appropriate imaging techniques (either small slice thickness, 3D sequences or combination of different slice orientations), applicator characterization
Interfraction/Intrafraction changes between imaging and dose delivery	7⁎	For one treatment plan per applicator insertion and measures to detect major variations for subsequent fractions
Total dosimetric uncertainty (*k* = 1)	**13**	For treatment delivered with the same BT source

⁎ Estimated value based on expert discussion.

**Table 4:** Example 4 – LDR ^125^I sources for permanent prostate BT.

Category	Typical level (%)	Assumptions
Source strength	3	PSDL traceable calibrations
Treatment planning	4	Reference data with the appropriate bin width
Medium dosimetric corrections	5	No consideration is given for calcifications or their composition in the patient
Inter-seed attenuation	4	An advanced dose calculation formalism may indicate source models and orientations cause the largest effects
Treatment delivery imaging	2	US QA performed according to AAPM TG-128
Target contouring uncertainty	2	Using CT or CT + T2 imaging
Anatomy changes between dose delivery and post-implant imaging	7⁎	Post-implant imaging using CT, with a scalar correction factor for edema correction
Total dosimetric uncertainty (*k* = 1)	**11**	For treatment delivered without excreted seeds

⁎ Estimated value based on expert discussion.

**Table 5:** Example 5 – HDR ^192^Ir source for temporary prostate BT.

Category	Typical level (%)	Assumptions
Source strength	2	PSDL traceable calibrations
Treatment planning	3	Reference data with the appropriate bin width
Medium dosimetric corrections	1	Full scatter conditions in the pelvic region and for the prostate location are assumed
US-based Treatment planning and delivery: Catheter reconstruction and source positioning accuracy	2	Assuming usage of dedicated catheter reconstruction tools (catheter free-length measurement based methods) for an accurate (0.7 mm) reconstruction of catheter tip and 1.0 mm source positioning accuracy by the afterloader for straight catheters and transfer tubes
US-based 2D and 3D-imaging overall effect	2	US QA performed according to AAPM TG-128 report
Changes of catheter geometry relative to anatomy between intraoperative treatment planning and intraoperative treatment delivery	2	Assuming that new image acquisition and treatment plan calculation is done always before each fraction. It is also required that no manipulation of the implant and anatomy occurs, as it is the case when removing/manipulating the US-probe or moving the patient from the operation table before treatment delivery
Target contouring uncertainty	2	Using CT or CT + T2 imaging
Total dosimetric uncertainty (*k *= 1)	**5**	For treatment delivery without patient movement and changes in the lithotomic set-up and with the US probe at the position of the acquisition (transversal plane at the prostate base)
